# Exploration of the Risk Factors of Generalized and Central Obesity among Adolescents in North Lebanon

**DOI:** 10.1155/2017/2879075

**Published:** 2017-09-11

**Authors:** Germine El-Kassas, Fouad Ziade

**Affiliations:** ^1^Nutrition & Dietetics Department, Faculty of Health Sciences, Beirut Arab University, Tripoli 1301, Lebanon; ^2^Faculty of Public Health, Lebanese University, Tripoli 1301, Lebanon

## Abstract

Adolescents' obesity is an emerging public health problem globally and in the Arab countries. Alarming rates of overweight/obesity have been rising progressively in Lebanon. However, the risk factors for the development of adolescents' obesity have not yet been thoroughly explored in North Lebanon. To determine the dietary and lifestyle risk factors associated with generalized and central obesity among adolescents living in Tripoli, a cross-sectional survey was conducted including a representative sample of 311 students aged 11–16 years from both sexes chosen from public and private schools in Tripoli. Data were collected using a standardized questionnaire to determine sociodemographic characteristics, dietary patterns, and physical activity and sedentary behaviors. Body mass index (BMI) was evaluated using the Center for Disease Control BMI for age percentiles. Central obesity was assessed using both waist-to-height ratio and gender-specific waist circumference for age indices. Multiple logistic regression analysis revealed that skipping breakfast and physical inactivity were the most significant independent risk factors associated with both generalized and central obesity. In addition, higher screen time and male gender were associated with increased risk for generalized and central obesity, respectively. Intervention strategies to prevent the development of obesity should be implemented among adolescent students to encourage regular breakfast intake and adopting healthy dietary and lifestyle behaviors.

## 1. Introduction

Adolescence obesity is a growing global epidemic with a large variation in secular trends across countries [1]. During the past decades, the prevalence of overweight or obesity in school children and adolescents had been doubled or tripled throughout several developed and developing countries [2]. Studies in some Arab countries and in the Middle East have revealed that the prevalence of overweight and obesity has reached dramatic rates and is considered as a major public health problem [3, 4].

It has been suggested that a “westernized” lifestyle of excessive energy intake and sedentary behaviors partially explains the recent emergence of obesity among youth [5]. This was typically shown in Lebanon, a country that has experienced rapid nutrition transition from the traditional Mediterranean diet into the westernized pattern over the past decade [6]. Studies conducted between the years 1997 and 2009 showed a rise in the prevalence of obesity among Lebanese adolescents from 7.3% to 10.9% [7]. This indicates that the nutrition transition in Lebanon has been reflected into an alarming increase in prevalence of obesity among adolescents [7].

Accumulating evidence indicates that adolescent onset obesity increases the long-term morbidity and mortality [8]. Metabolic complications associated with obesity in childhood greatly increase the risk for type 2 diabetes, early cardiovascular diseases, hepatic disorders, and some types of cancer [8, 9]. Additionally, overweight and obesity affect the self-esteem of children and impair social development [10]. Longitudinal studies had suggested that adolescence obesity may track into adulthood as 70% of 10-year-old to 13-year-old obese children would become obese adults [11]. Moreover, growing evidence suggests that central adiposity independently of generalized obesity is more closely associated with cardiometabolic complications in childhood and adolescence [12, 13]. It is hypothesized that the long-term complications of obesity project an increasing public health burden especially in developing countries which may suffer consequently from the double burden of malnutrition. This in turn necessitates early detection and management of childhood and adolescence obesity at both the individual and the community levels [14].

It is well established that obesity is a complex multifactorial disease with multiple etiologies which includes some nonmodifiable risk factors like genetics and demographic factors; however, other factors like dietary and lifestyle behaviors can be targeted and modified [15]. Management of obesity in this age group implies early detection and modification of the underlying cause [16].

Several studies suggested that schools are a potential setting to adolescent population for promotion of healthy lifestyles and obesity prevention [17, 18]. On this context, identifying risk factors associated with overweight and obesity in school adolescents would help further implementation of the most relevant nutritional and health-promoting programs to reduce the future burden of overweight and obesity among young population in Lebanon. A growing evidence from studies across both developed and developing countries indicated that there is regional variation in the prevalence and the type of risk factors associated with obesity in the same country [19–23]. Review of the current available literature indicated that there is scarcity of data on the risk factors associated with adolescents' generalized and central obesity in North Lebanon. Therefore, this study was carried out aiming at investigating the risk factors associated with generalized overweight/obesity and central obesity among adolescent school students in Tripoli, the capital city of North Lebanon governorate.

## 2. Methods 

### 2.1. Study Design and Participants

Through a cross-sectional study design, a survey was conducted in Tripoli during the period between October and December 2014. The study participants were students aged 11–16 years corresponding to grades 7 to 9 in the selected schools.

### 2.2. Sampling Procedure

The calculated minimal sample size for the study was 292 students based on an anticipated population proportion of 12.8% [7], with a margin of error of 5% at 99% confidence level (CI). Then the sample was increased by 10% to make up for any missing data. The study was approved by the Institutional Review Board at Beirut Arab University. The schools in Tripoli were stratified into public and private. Four intermediate level mixed (boys and girls) schools divided into public and private were selected randomly using a cluster random sampling technique. One class from each grade in these schools was further selected randomly to be included in the study. Directors of these schools were contacted, and all of them agreed to participate in the current study. Parents of students in the targeted schools were informed by the school principles to have their approval for their siblings to participate in the study. None of the students refused to participate. The students' school health profiles were reviewed. Inclusion criteria implied students who were present on the days of data collection and were found to be free from any medical condition and showed their oral consent to participate. The exclusion criteria included those who were suffering from any physical disabilities or chronic metabolic disease and those who were on long-term medication for more than six months. Those who were absent on the days of data collection were excluded.

### 2.3. Data Collection

A structured anonymous interview questionnaire based on previously published instrument which has been standardized and validated to be used among adolescent school students was used [24, 25]. The questionnaires were applied in the classrooms by trained researchers who had participated in previous training to standardize the data collection procedures. Each student was interviewed separately to ensure privacy. A series of regular visits to schools were conducted to ensure high coverage rate and maximize the number of students participating in the study. The questionnaire included questions to assess the sociodemographic characteristics, dietary and food intake patterns, and physical activity and lifestyle behaviors followed by anthropometric measurements.

### 2.4. Measures

#### 2.4.1. General and Sociodemographic Characteristics

Questions inquiring about age, gender, the type of current residence, educational level, and occupation of parents were asked to define the general and sociodemographic characteristics of the study sample. The educational level of both father and mother was categorized as high for those who completed their university studies or higher and low for those having lower educational level than the university level.

#### 2.4.2. Anthropometric Measurements

Anthropometric measurements including weight, height, and waist circumference (WC) were assessed by well-trained researchers using standardized techniques [26] and calibrated scales. Standing height was measured to the nearest 0.1 cm without shoes, using a stadiometer. Participants wearing light clothes were weighed to the nearest 0.1 kg, on an electronic scale, which was first calibrated using a standard weight, and rechecked daily. WC measurements were done using a nonstretchable measuring tape with centimeter and millimeter markings at the level of the umbilicus to the nearest 0.1 cm with the subject standing and after normal expiration. All measurements were taken twice and the average of the 2 values was recorded.

Body mass index (BMI) was calculated using the following formula: body weight (Kg)/height (m^2^). The degree of overweight/obesity was defined as having a BMI higher than the 85th percentile using the age- and sex-specific BMI growth charts developed by the United States Centers for Disease Control and Prevention (CDC) [27]. Central obesity was assessed by using two indices. The first was WC measurement evaluation. The cut-off points suggested by Taylor et al. [28] were used to identify cases of central obesity. The selection of this assessment reference was based on the results presented in a study conducted to evaluate the sensitivity and specificity of two reference charts for WC in children and adolescents [29]. Also the waist-to-height ratio (WHtR) which is a simple noninvasive index for evaluation of central adiposity and has higher sensitivity in the prediction of cardiometabolic risk in children and adolescents than BMI was used for detecting central obesity [13, 30]. The WHtR index for central obesity was calculated by dividing WC by height, both measured in centimetres, and the cut-off point of ≥0.5 was used to identify children with elevated WHtR [13, 31].

#### 2.4.3. Dietary Intake Assessment

The dietary and food intake patterns including the regularity of meal consumption, regular breakfast intake, number of meals, number and type of snacks, types and amount of drinks consumed, and frequency of fast food consumption were assessed.

#### 2.4.4. Physical Activity and Lifestyle Variables

The physical activity questionnaires for older children (PAQ-C) and adolescents (PAQ-A) which have been validated to evaluate the physical activity levels among children and adolescents were used to assess the physical activity level of the participants [25, 32]. PAQ-C was used to assess the physical activity level for participants below the age of fourteen, while PAQ-A was used to assess the physical activity level of participants at the age of fourteen or more [25, 32]. PAQ-C includes 10 items and PAQ-A includes 9 items to assess the physical activity throughout the week. The last item in both questionnaires is used to assess students having unusual activities during the last week and is not included in the score. Based on the mean score of items from 1 to 9 for PAQ-C and items from 1 to 8 for PAQ-A, weekly frequency of activities of students was classified into two categories: active (high) and inactive (low). For evaluation of sedentary behaviors, a maximal cut-off value for total screen time equivalent to 2 hours per day, recommended by the American Academy of Pediatric guidelines, was used [30].

### 2.5. Statistical Analysis

To fulfill the aims of the study, specific statistical methods were used to analyse various sociodemographic variables among private and public school students and to analyse anthropometric, lifestyle, and dietary behaviors based on gender. Frequencies, means, and standard deviation (SD) were used to describe anthropometric characteristics and dietary and lifestyle behaviors. Chi-square test and Student's *t*-test were used to compare proportions and means, respectively. When more than 20% of the cells have expected count less than 5, correction for chi-square was conducted using Monte Carlo correction. Multivariate logistic regression analysis was carried out to examine the association of generalized overweight/obesity, elevated WC, and elevated WHtR as the dependent variables with baseline sociodemographic, lifestyle, and dietary characteristics as covariates. Based on univariate analysis results, variables which were found to have significant associations with generalized overweight/obesity and/or central obesity up to 15% (*p* = 0.15) were included in the analysis [33]. These variables are sex, school type, father's education, mother's education, father's occupation, number of family members, regular breakfast consumption, type of drink between meals, intake of fast food/week, screen time, and physical activity level. These covariates were entered simultaneously, each as an independent variable. *p* value of <0.05 was considered statistically significant. All analyses were performed using the Statistical Package for Social Sciences (SPSS) (version 20, Armonk, NY, USA).

## 3. Results

### 3.1. Characteristics of the Subjects

Table 1 compares between the sociodemographic variables among adolescents in private and public schools. A total of 311 adolescent school students with complete data were included in the analysis. Table 1 demonstrates that the mean ages (13.28 ± 0.93 years versus 13.96 ± 1.30 years, *p* < 0.001) as well as the mean of the number of family members (5.31 ± 1.03 members versus 5.62 ± 1.19 members, *p* = 0.014) of students in private schools compared to those in public schools, respectively, showed a statistically significant difference. The sex distribution revealed a statistically significant difference between private and public school participants (55.6% male participants in private schools versus 10.8% male participants in public schools, *p* < 0.001). The table also shows that 34% of private school students' fathers versus 3% only of public schools students' fathers had high level of education (*p* < 0.001). Moreover, 47.2% of private schools students' mothers versus 7.8% only of public schools students' mothers had high level of education (*p* < 0.001). The table also shows a statistically significant difference between both the father's occupation (50% versus 32.9% of fathers work as employee or professional, *p* < 0.001) and the mother's occupation (34% versus 10.8% of mothers work as employee or professional, *p* = 0.001) of private and public school students, respectively. In addition, the results shows that 82.6% of private school students compared to 98.2% of public schools students live in urban areas with a statistically significant difference (*p* < 0.001).

### 3.2. Anthropometric Measurements

Table 2 compares the anthropometric characteristics based on the gender of the study participants in public and private schools. The results of this study showed that the overall prevalence rate of generalized overweight/obesity was 32.2% and those of central obesity were 41.8% and 38.3% (evaluated by WC and WHtR indicators, resp.). Based on BMI classification, the prevalence of generalized overweight/obesity was significantly more common among male adolescent students compared to females (40.8% versus 28.2%, resp., *p* = 0.027). As regards central obesity, gender-based statistically significant differences were also detected using both WC and WHtR indicators (*p* < 0.001  and  *p* = 0.001, resp.) as shown in Table 2.

### 3.3. Dietary Intake Patterns and Behaviors

The meal patterns and dietary intake habits were compared by gender in Table 3. The table shows that about one-quarter (22.4%) of the male adolescent students consumed only 2 main meals/day compared to more than one-third (36.6%) of the female adolescent students with a statistically significant difference between them (*p* = 0.003). Concerning the intake of snacks, nearly half of the male (49%) and female adolescent students (50.2%) reported regular intake of two snacks daily with no statistically significant difference between them. On the other hand, a statistically significant (*p* = 0.024) higher proportion of the male students (65.3%) reported regular daily intake of breakfast compared to only 51.6% of the female students.

As shown in Table 3, both male and female adolescents showed unhealthy dietary habits and choices as regards the type of snacks, type of drinks between meals, daily intake of milk or yoghurt, and time lapse before sleep with no statistically significant difference. Males tend to eat more frequently at fast food restaurants as compared to females (34.7% of males versus 30.0% of females eat 2-3 times/week) but the difference did not reach statistical significance.

### 3.4. Physical Activity and Lifestyle Behaviors

Table 4 describes the physical activity level and sedentary behaviors of adolescent students based on gender. Evaluation of the physical activity showed that the majority of the studied sample (82.3%) were physically inactive with statistically significant (*p* < 0.001) higher prevalence of physical inactivity among females (88.3%) compared to males (69.4%).

The table also shows that more than half of male (58.2%) and female (53.1%) adolescent students spend more than 2 hours either watching TV or using computer or other screens for nonstudying purposes with no statistically significant difference between the two genders.

### 3.5. Associations of Sociodemographic, Lifestyle, and Dietary Factors with Generalized Overweight/Obesity, Elevated Waist-to-Height Ratio, and Elevated Waist Circumference

Multivariate regression analysis revealed that skipping breakfast (OR: 5.508; 95% CI: 3.168–9.575), low physical activity (OR: 2.199; 955 CL: 1.141–4.240), and increased screen time (OR: 1.562; 95% CI: 1.155–2.112) were associated with significantly higher odds for the development of generalized overweight/obesity as shown in Table 5. Concerning central obesity, multivariate regression analysis showed that skipping breakfast was associated with statistically significant higher odds of having both elevated WHtR and elevated WC [(OR: 2.339; 95% CI: 1.401–3.904) and (OR: 2.653; 95% CI: 1.594–4.414)]. Male gender exhibited significantly higher odds for increasing central adiposity as detected by WHtR (OR: 1.921; 95% Cl: 1.108–3.330). In addition, low physical activity was associated with significantly higher odds of having elevated WC (OR: 1.732; 95% CI: 1.001–3.006). In contrast, higher consumption of sugar-sweetened beverages was associated with lower odds of having both elevated WHtR and elevated WC [(OR: 0.539; 95% CI: 0.311–0.935) and (OR: 0.513; 95% CI: 0.297–0.885); see Table 5].

## 4. Discussion

The present study investigated the dietary habits and lifestyle factors associated with generalized overweight/obesity and central obesity among adolescents living in an urban city in North Lebanon (Tripoli) through a cross-sectional survey of a school-based sample including both public and private schools which, as far as is known, have not been studied before. The results of the present study demonstrated several significant associations of risk factors with the development of generalized overweight/obesity and central obesity including skipping breakfast, male gender, physical inactivity, and high screen time. However, our findings did not detect significant associations between any of the sociodemographic factors studied and either of general or central obesity. It has to be noted that comparison of data across different studies is somewhat challenging because of the inconsistency in the sampling techniques and design, variable age ranges, and different reference cut-off criteria.

The findings of the present study showed that the overall prevalence of generalized overweight/obesity among the studied sample was about one-third (32.2%). When comparing the present findings by data reported using the CDC cut-offs from different countries, similar figures were reported among adolescents in Egypt [34] and Argentina (35.5%) [35], lower figures were reported in Turkey (15.7%) [36], and much lower figures were reported from Iran (6.4%) [37]. On the other hand, much higher prevalence rate (52%) was reported in the United States by NHANES 2011 [38]. In Lebanon, data reported from a cross-sectional survey conducted by Chacar and Salameh in 2007 among adolescents aged 11–18 years in public schools indicated overall lower prevalence rate equal to 27.1% of combined overweight and obesity [39]. The relative inconsistency in the prevalence rates between our findings and the findings of Chacar and Salameh could be attributed to the fact that about half (46.3%) of the adolescents included in the current study were from private schools whose students are known to be of higher socioeconomic level than those of public schools.

Differences encountered in the prevalence of generalized overweight and obesity rates across countries may be attributed to sociocultural factors, environmental, physical activity levels, and nutritional knowledge and health awareness in these diversities of study samples across countries^.^ [40]. Nevertheless, it is difficult to reach a concrete conclusion, since we are comparing regional data from an urban city with national data, which included both urban and rural areas. Therefore, it is necessary in any national information on obesity to show the regional differences in the obesity proportion.

The prevalence of central obesity in Lebanon had followed an alarming increasing trend over time, which has been paralleled by a rise in the prevalence of metabolic diseases such as hypertension, diabetes, and hyperlipidemia [41]. Published data revealed increasing rates of elevated WHtR from 19.1 to 21.3% between the years 1997 and 2009 among 6–19-year-old children and adolescents [7]. The current findings showed alarming high prevalence of abdominal obesity as assessed by both elevated WHtR and elevated WC indicators, especially among males. Recently published data from a household-based national survey conducted in 2009 in Lebanon reported lower rates of elevated WHtR within the age group of 12–19 years (26.2% and 15.6% among males and females, resp.) compared to the present findings [42]. The differences encountered from our findings could be attributed to the urban nature of the present sample where westernized dietary habits and lifestyle commonly associated with higher prevalence of general and central obesity are highly adopted among adolescents [22, 23]. Additionally, the present study is more recent (four years later), indicating the ongoing progressive rising trend of central obesity as previously reported [7, 41]. Comparable results to the present findings were reported by a school-based multicentre cross-sectional study in Saudi Arabia [23] which showed that 35.9% and 30.3% of male and female adolescents, respectively, were suffering from central obesity as indicated by elevated WHtR. Lower prevalence rates (based on WHtR indicator) were seen in Iran (20.84%) [37] and Egypt (16.7%) [34]. Based on elevated WC indicator, the present results were comparable with data reported in Brazil (32.7%) [43] (using the same reference standards) but were higher than those previously reported in Lebanon (using different reference standards) [42].

Statistically significant higher prevalence (*p* = 0.027) of generalized overweight/obesity among males compared to females was detected in the present study. Similar findings (using the CDC standards as in the present study) were seen in Jordan (29% among males versus 19.4% among females), Kuwait (47.5% among males versus 38.8% among females), and United Arab of Emirates (33.6% among males versus 18.5% among females) [44]. In Lebanon, a study conducted among private school children and adolescents had also shown that the prevalence of obesity among males was almost triple that among females [45]. In contrast, higher rate of overweight and obesity among Saudi female adolescents living in southwestern Saudi Arabia compared to that among male adolescents living in the same area had been reported (29.4% among females versus 23.2% among males) [21]. Gender-based differences were detected as well by central obesity indicators (elevated WHtR and elevated WC) in the current study. Published data concerning gender differences in the prevalence of central obesity indicated controversial results. However, results of a systematic review on central obesity among adolescents in both developed and developing countries concluded that it is not clear what sex has a higher proportion of central obesity and there is no consensus in the literature about the criteria to be used [46]. The lower prevalence of generalized overweight/obesity and central obesity among females compared to males in the present study may be explained by the finding that young females are more concerned about their body shape and weight status than males [47].

Lifestyle and behavioral changes during adolescence can result in alterations in food habits as adolescents tend to skip daily main meals and have a relatively low vitamin and mineral intakes [48]. Breakfast consumption has been reported to be an important determinant of adolescent overweight and obesity [49]. The underlying suggested mechanisms included the association of regular breakfast consumption with a reduction in dietary fat, reduced impulsive snacking, lower cholesterol, and lower body weight [50]. In contrast, skipping breakfast may lead to excess hunger, rebound overeating, and consumption of larger portion sizes and consequent obesity [51, 52]. Skipping breakfast was found to be the strongest predictive risk factor for the development of generalized overweight/obesity and central obesity in the present sample. This was in line with data reported by Saudi, Swedish, and Chinese adolescent students [22, 53, 54].

In Lebanon, school children and adolescents are adopting a westernized diet known for excessive amounts of saturated fats and refined carbohydrates, while abandoning the Mediterranean diet that is rich in whole-grain cereals, vegetables, and fruits [41]. This was clearly shown in the present study, where most of the adolescents from both sexes reported unhealthy meal and snacking patterns, high intake of fast foods, and high consumption of sugar-sweetened beverages including soft drinks. The higher consumption of sugar-sweetened beverages currently reported by the adolescent students, unexpectedly, was significantly associated with decreasing the risk for the development of central obesity as assessed by both elevated WHtR and WC indicators. These findings were in agreement with data reported by a large prospective cohort study among a diverse population of 4746 adolescents from various socioeconomic and ethnic backgrounds in 31 public middle and high schools in Minnesota [55]. In addition, a risk assessment study of the relationship of regular carbonated soft drink (RCSD) consumption in schools and body mass index showed no impact on BMI by removing consumption of RCSD in schools [56]. In contrast, the conclusions of a systematic review documented a positive association between greater intakes of sugar-sweetened beverages and obesity in children and adults [57]. These conflicting findings may be related to error or bias in self-report of dietary intake data which has been revealed by validation studies using objective measures of dietary intake especially among overweight/obese children and adolescents [58, 59]. This bias in self-reported dietary intake data may in turn mask associations between diet and weight status [60]. Moreover, the association between sugar-sweetened beverages and weight gain may be changed after controlling for total energy intake [61].

Increased frequency of fast foods consumption has been correlated to obesity development among adolescents [62]. In contrast, other researchers could not detect an association between fast food consumption and obesity in the age group between 11 and 19 years [42, 63]. The latter findings were consistent with reported data in the present study. A possible explanation might be that the portion size of fast foods, which was not accounted for in this study, may be a confounding factor and could influence the association between the frequency of fast food consumption and obesity.

Lifestyle factors such as sedentary behaviors and physical inactivity may play a crucial role in creating an obesogenic environment [64]. Evaluation of the physical activity behaviors of students participating in the present study identified that physical inactivity was significantly associated with both generalized overweight/obesity and central obesity. The current findings are consistent with the growing evidence showing that physical inactivity is a leading factor in obesity during childhood and adolescence [65]. Consistent findings were previously reported among adolescents in both developed and developing countries [42, 66, 67]. In contrast, some others failed to show a significant association of physical inactivity with obesity development [63, 68], while others reported a protective significant association against obesity with vigorous physical activity only [22, 69]. These controversies suggest that different populations and variable sampling techniques may have variable outcomes. A mounting body of evidence shows that sedentary behaviors are associated with adverse health outcomes in a way that seems to be different from those attributed to the lack of physical activity [68–70]. Sedentary behaviors and physical activity are considered to be independently associated with adiposity and metabolic risk. The association between sedentary behaviors like TV viewing and clustered metabolic risk is mediated by adiposity, whereas physical activity is associated with individual and clustered metabolic risk indicators independently of obesity [68]. In the present study, screen viewing for more than two hours daily was significantly associated with increased risk of generalized overweight/obesity among adolescents of the present sample. Consistent findings with the present study documented significant associations between sedentary behaviors and obesity development among adolescents in Jordan and Bangladesh who spent more than 2 and 4 hours daily on sedentary behaviors, respectively [70, 71].

## 5. Conclusions

The current research highlights some significant associations of several modifiable dietary and lifestyle risk factors associated with the development of alarming rates of general and central obesity among adolescents living in an urban city in North Lebanon. These data provide insights for policy makers to consider reversing the obesity epidemic rates as a national public health priority. Primary prevention and control measures to cut the vicious cycle of obesity and obesity-related metabolic diseases should be implemented. These measures include elaborating cultural-specific comprehensive educational programs in schools to reduce sedentary behaviors, enhance physical activity, and encourage adoption of healthy dietary patterns and choices such as regular breakfast consumption and healthy snacking and beverage intake. In addition, performing periodic surveys on national or subnational levels may provide data necessary for formulation of specific control measures. Finally, it is a must for all parents, educators, and health professionals to integrate efforts aiming to reverse the upward trends of generalized and central obesity among adolescents, which could negatively affect the health of the upcoming generations.

## Figures and Tables

**Table 1:** General characteristics of the study sample.

	School type				Test of sig.	*p*
	Private (*N*. = 144)		Public (*N*. = 167)			
	*N*.	%	*N*.	%		
*Age (years)*						
Min.–Max.	12.0–16.0		11.0–16.0		*t* = 5.359^*∗*^	<0.001^*∗*^
Mean ± SD	13.28 ± 0.93		13.96 ± 1.30			

*Sex *						
Male	80	5.6	18	10.8	*χ* ^2^ = 71.837^*∗*^	<0.001^*∗*^
Female	64	44.4	149	89.2		

*Educational level of father*						
Illiterate	5	3.5	8	4.8	*χ* ^2^ = 79.360^*∗*^	<0.001^*∗*^
Primary school	9	6.3	44	26.3		
Preparatory school	28	19.4	71	42.5		
Secondary school	29	20.1	23	13.8		
Above secondary	24	16.7	16	9.6		
University	49	34.0	5	3.0		

Low	95	66.0	162	97.0	*χ* ^2^ = 51.902^*∗*^	<0.001^*∗*^
High	49	34.0	5	3.0		

*Educational level of mother*						
Illiterate	3	2.1	5	3.0	*χ* ^2^ = 86.876^*∗*^	<0.001^*∗*^
Primary school	3	2.1	33	19.8		
Preparatory school	21	14.6	61	36.5		
Secondary school	20	13.9	35	21.0		
Above secondary	29	20.1	20	12.0		
University	68	47.2	13	7.8		

Low	76	52.8	154	92.2	*χ* ^2^ = 62.438^*∗*^	<0.001^*∗*^
High	68	47.2	13	7.8		

*Father's occupation*						
No regular job	2	1.4	8	4.8	*χ* ^2^ = 39.811^*∗*^	<0.001^*∗*^
Employee	52	36.1	52	31.1		
Technician	13	9.0	22	13.2		
Labor worker	10	6.9	44	26.3		
Merchant	25	17.4	23	13.8		
Professional	20	13.9	3	1.8		
Other	22	15.3	15	9.0		

Employee + professional	72	50.0	55	32.9	*χ* ^2^ = 9.321^*∗*^	0.002^*∗*^
Other	72	50.0	112	67.1		

*Mother's occupation*						
No regular job	82	56.9	143	85.6	*χ* ^2^ = 43.464^*∗*^	<0.001^*∗*^
Employee	42	29.2	14	8.4		
Technician	1	0.7	1	0.6		
Labor worker	0	0.0	4	2.4		
Merchant	6	4.2	1	0.6		
Professional	7	4.9	4	2.4		
Other	6	4.2	0	0.0		

Employee + professional	49	34.0	18	10.8	*χ* ^2^ = 24.728^*∗*^	<0.001^*∗*^
Other	95	66.0	149	89.2		

*Residence area*					*χ* ^2^ = 22.865^*∗*^	<0.001^*∗*^
Urban	119	82.6	164	98.2		
Rural	25	17.4	3	1.8		

*Number of family members*						
Min.–Max.	3.0–8.0		3.0–8.0		*t* = 2.465^*∗*^	0.014^*∗*^
Mean ± SD	5.31 ± 1.03		5.62 ± 1.19			

*χ* ^2^: Chi square test; *t*: Student's *t*-test; sig.: significance. ^*∗*^Statistically significant at *p* ≤ 0.05. *N*.: number.

**Table 2:** Anthropometric assessment.

	School type												Total sample (*N*. = 311)	
	Male (*N*. = 98)				Female (*N*. = 213)				Total (*N*. = 311)					
	Private		Public		Private		Public		Male		Female			
	*N*.	%	*N*.	%	*N*.	%	*N*.	%	*N*.	%	*N*.	%	*N*.	%
*Body mass index*														
Underweight/normal	48	60.0	10	55.6	47	73.4	106	71.1	58	59.2	153	71.8	211	67.8
Overweight/obese	32	40.0	8	44.4	17	26.6	43	28.9	40	40.8	60	28.2	100	32.2

*χ*^2^ (*p*)	0.120 (0.729)				0.117 (0.733)				4.921^*∗*^ (0.027^*∗*^)					

*Waist circumference *														
Normal	34	42.5	12	66.7	37	57.8	98	65.8	71	49.3	110	65.9	181	58.2
Central obesity	46	57.5	6	33.3	27	42.2	51	34.2	73	50.7	57	34.1	130	41.8

*χ*^2^ (*p*)	3.446 (0.063)				1.222 (0.269)				8.719^*∗*^ (<0.001^*∗*^)					

*Waist-to-height ratio *														
Normal	35	43.8	12	66.7	40	62.5	105	70.5	75	52.1	117	70.1	192	61.7
Central obesity	45	56.3	6	33.3	24	37.5	44	29.5	69	47.9	50	29.9	119	38.3

*χ*^2^ (*p*)	3.092 (0.079)				1.308 (0.253)				10.578^*∗*^ (0.001^*∗*^)					

*χ* ^2^: Chi square test. ^*∗*^Statistically significant at *p* ≤ 0.05. *N*.: number.

**Table 3:** Dietary intake patterns and behaviors.

Variables		Total (*N*. = 311)		Male (*N*. = 98)		Female (*N*. = 213)		*χ* ^2^	*p*
		*N*.	%	*N*.	%	*N*.	%		
Number of main meals	One	14	4.5	6	6.1	8	3.8	13.840^*∗*^	0.003^*∗*^
	Two	100	32.2	22	22.4	78	36.6		
	Three	180	57.9	59	60.2	121	56.8		
	More than three	17	5.5	11	11.2	6	2.8		

Number of snacks	Zero	7	2.3	1	1.0	6	2.8	1.797	^MC^ *p* = 0.786
	One	90	28.9	30	30.6	60	28.2		
	Two	155	49.8	48	49.0	107	50.2		
	Three	36	11.6	10	10.2	26	12.2		
	More than three	23	7.4	9	9.2	14	6.6		

Regular daily breakfast consumption	Yes	174	55.9	64	65.3	110	51.6	5.084^*∗*^	0.024^*∗*^
	No	137	44.1	34	34.7	103	48.4		

Snacks based mainly on	No snacks	5	1.6	4	4.1	1	0.5	9.306	^MC^ *p* = 0.143
	Fruit/fruit juice/yogurt	12	3.9	6	6.1	6	2.8		
	Biscuits	26	8.4	6	6.1	20	9.4		
	Fried potatoes	11	3.5	2	2.0	9	4.2		
	Sweets/chocolate/cake	26	8.4	7	7.1	19	8.9		
	Combinations (potato chips + chocolate)	191	61.4	58	59.2	133	62.4		
	Others	40	12.9	15	15.3	25	11.7		

Type of usually consumed drinks	Water	103	33.1	41	41.8	62	29.1	5.146	^MC^ *p* = 0.141
	Sugar-sweetened beverages	165	53.1	44	44.9	121	56.8		
	Fresh fruit juice	39	12.5	12	12.2	27	12.7		
	Milk and milk shakes	4	1.3	1	1.0	3	1.4		

Regular daily consumption of 1 glass of milk or 1 cup of yogurt	Never	95	30.5	25	25.5	70	32.9	2.839	0.417
	Sometimes	120	38.6	37	37.8	83	39.0		
	Often	33	10.6	12	12.2	21	9.9		
	Always	63	20.3	24	24.5	39	18.3		

Time lapse between last meal and going to bed	Less than 2 hours	186	59.8	64	65.3	122	57.3	1.800	0.180
	More than 2 hours	125	40.2	34	34.7	91	42.7		

Frequency of fast food meal consumption/week	0-1	191	61.4	54	55.1	137	64.3	3.370	0.185
	2-3	98	31.5	34	34.7	64	30.0		
	More than 3	22	7.1	10	10.2	12	5.6		
	Less than 3	289	92.9	88	89.8	201	94.4	2.133	0.144
	More than 3	22	7.1	10	10.2	12	5.6		

*χ* ^2^: Chi square test; MC: Monte Carlo test. ^*∗*^Statistically significant at *p* ≤ 0.05. *N*.: number.

**Table 4:** Physical activity and sedentary behaviors.

Variables	Total		Male (*N*. = 98)		Female (*N*. = 213)		*χ* ^2^	*p*
	*N*.	%	*N*.	%	*N*.	%		
*Physical activity assessment*								
Active	55	17.7	30	30.6	25	11.7	16.426^*∗*^	<0.001^*∗*^
Inactive	256	82.3	68	69.4	188	88.3		
*Screen time*								
Less than 2 hours/day	141	45.3	41	41.8	100	46.9	0.708	0.400
More than 2 hours/day	170	54.7	57	58.2	113	53.1		

*χ* ^2^: Chi square test. ^*∗*^Statistically significant at *p* ≤ 0.05. Sig.: significance; *N*.: number.

**Table 5:** Associations of sociodemographic, lifestyle, and dietary factors with generalized overweight/obesity, elevated waist to height ratio, and elevated waist circumference.

	Generalized overweight/obesity		Elevated waist-to-height ratio		Elevated waist circumference	
	Sig.	OR (95% CI)	Sig.	OR (95% CI)	Sig.	OR (95% CI)
*Sex *						
Female®		1.00		1.00		1.00
Male	0.07	1.77 (0.96–3.26)	0.02^*∗*^	1.92 (1.11–3.33)	0.07	1.68 (0.97–2.90)
*School type*						
Private®		1.00		1.00		1.00
Public	0.91	0.96 (0.47–1.97)	0.32	1.39 (0.73–2.63)	0.09	1.72 (0.91–3.25)
*Father's education *						
High®		1.00		1.00		1.00
Low	0.52	0.93 (0.73–1.17)	0.12	1.18 (0.96–1.46)	0.51	1.07 (0.87–1.32)
Mother*'s* education						
High®		1.00		1.00		1.00
Low	0.80	1.03 (0.82–1.30)	0.82	1.024 (0.83–1.26)	0.32	1.11 (0.91–1.36)
*Father's occupation *						
Professional®		1.00		1.00		1.00
Other	0.21	0.68 (0.37–1.24)	0.24	1.38 (0.80–2.38)	0.64	1.13 (0.67–1.93)
*Number of family members*	0.31	0.75 (0.43–1.31)	0.20	0.72 (0.44–1.18)	0.54	0.86 (0.53–1.40)
*Regular breakfast consumption*						
Yes®		1.00		1.00		1.00
No	<0.01^*∗*^	5.51 (3.17–9.58)	0.01^*∗*^	2.34 (1.40–3.90)	<0.01^*∗*^	2.65 (1.59–4.41)
*Type of drink between meals*						
Water®		1.00		1.00		1.00
Fresh fruit juice	0.21	0.53 (0.20–1.44)	0.90	1.05 (0.47–2.36)	0.59	1.25 (0.56–2.76)
Milk and milk shakes	0.85	1.25 (0.12–13.06)	0.88	0.84 (0.09–7.68)	0.77	0.73 (0.08–6.42)
Sweetened beverages	0.18	0.66 (0.36–1.21)	0.03^*∗*^	0.54 (0.31–0.94)	0.02^*∗*^	0.51 (0.30–0.89)
*Fast food meal/week*						
<3 times®		1.00		1.00		1.00
>3 times	0.38	1.56 (0.58–4.20)	0.77	0.87 (0.37–2.25)	0.59	0.77 (0.30–1.98)
*Screen time*						
Less 2 hours/day®		1.00		1.00		1.00
More 2 hours/day	0.01^*∗*^	1.56 (1.16–2.11)	0.07	1.29 (0.98–1.70)	0.13	1.24 (0.94–1.62)
*Physical activity level *						
Active®		1.00		1.00		1.00
Inactive	0.02^*∗*^	2.20 (1.14–4.24)	0.14	1.52 (0.88–2.62)	0.04^*∗*^	1.73 (1.01–3.0)

^*∗*^Statistically significant at *p* ≤ 0.05. Sig.: significance; ®: reference; *N*.: number.

## Additional Points

The findings of the present study should be seen in light of their strengths and limitations. The main strength of the present study is investigating the factors associated with obesity and central obesity across both public and private schools through a statistically representative sample of adolescent population in Tripoli, the capital city of North Lebanon, for the first time. We used two indicators for detection of central obesity of known sensitivity. In addition, the physical activity questionnaire used in this study is valid and reliable. Furthermore, data collection was through interview and not through self-reported questionnaires which could minimize the bias of self-reported data. This study has few limitations. Firstly, the cross-sectional study design does not allow disclosing trends of change in adiposity status and energy balance related behaviors in the same children. Secondly, the study had been conducted during a period of instabilities which may have resulted in the high rates of absentees, especially among males in public schools, leading to minimizing the proportion of male participants in the study. Thirdly, the use of international cut-offs due to unavailability of national age-specific BMI percentiles can be regarded as another limitation.
